# The Water–Energy–Food Nexus as a Tool to Transform Rural Livelihoods and Well-Being in Southern Africa

**DOI:** 10.3390/ijerph16162970

**Published:** 2019-08-18

**Authors:** Tafadzwanashe Mabhaudhi, Luxon Nhamo, Sylvester Mpandeli, Charles Nhemachena, Aidan Senzanje, Nafisa Sobratee, Pauline Paidamoyo Chivenge, Rob Slotow, Dhesigen Naidoo, Stanley Liphadzi, Albert Thembinkosi Modi

**Affiliations:** 1 Centre for Transformative Agricultural and Food Systems, School of Agricultural, Earth and Environmental Sciences, University of KwaZulu-Natal, P. Bag X01, Scottsville, Pietermaritzburg 3209, South Africa; 2 International Water Management Institute, Southern Africa (IWMI-SA), 141 Creswell Street, Silverton, Pretoria 0184, South Africa; 3 Water Research Commission of South Africa, 4 Daventry Street, Lynnwood Manor, Pretoria 0081, South Africa; 4 School of Environmental Sciences, University of Venda, Private Bag X 5050, Thohoyandou 0950, South Africa; 5 School of Engineering, University of KwaZulu-Natal, P. Bag X01, Scottsville, Pietermaritzburg 3209, South Africa; 6 School of Life Sciences, University of KwaZulu-Natal, P. Bag X01, Scottsville, Pietermaritzburg 3209, South Africa; 7 International Rice Research Institute (IRRI), DAPO Box 7777, 1301 Metro Manila, Philippines; 8 Department of Genetics, School of Genetics, Evolution & Environment, University College, London WC1E 6BT, UK

**Keywords:** climate change, livelihoods, innovation, human well-being, WEF nexus, adaptation, health

## Abstract

About 60% of southern Africa’s population lives in rural areas with limited access to basic services and amenities such as clean and safe water, affordable and clean energy, and balanced and nutritious diets. Resource scarcity has direct and indirect impacts on nutrition, human health, and well-being of mostly poor rural communities. Climate change impacts in the region are manifesting through low crop yields, upsurge of vector borne diseases (malaria and dengue fever), and water and food-borne diseases (cholera and diarrhoea). This study applied a water–energy–food (WEF) nexus analytical livelihoods model with complex systems understanding to assess rural livelihoods, health, and well-being in southern Africa, recommending tailor-made adaptation strategies for the region aimed at building resilient rural communities. The WEF nexus is a decision support tool that improves rural livelihoods through integrated resource distribution, planning, and management, and ensures inclusive socio-economic transformation and development, and addresses related sustainable development goals, particularly goals 2, 3, 6 and 7. The integrated WEF nexus index for the region was calculated at 0.145, which is marginally sustainable, and indicating the region’s exposure to vulnerabilities, and reveals a major reason why the region fails to meet its developmental targets. The integrated relationship among WEF resources in southern Africa shows an imbalance and uneven resource allocation, utilisation and distribution, which normally results from a ‘siloed’ approach in resource management. The WEF nexus provides better adaptation options, as it guides decision making processes by identifying priority areas needing intervention, enhancing synergies, and minimising trade-offs necessary for resilient rural communities. Our results identified (i) the trade-offs and unintended negative consequences for poor rural households’ livelihoods of current silo approaches, (ii) mechanisms for sustainably enhancing household water, energy and food security, whilst (iii) providing direction for achieving SDGs 2, 3, 6 and 7.

## 1. Introduction

Water, energy and food are vital resources for human well-being, poverty reduction and sustainable development [1,2]. Demand for these resources is projected to increase significantly in the near future due to population growth and migration, economic development, international trade, urbanisation, diversifying diets, cultural and technological changes, as well as climate variability and change [3]. These current societal megatrends, coupled with environmental, technological, economic and demographic changes, continue exerting pressure on already scarce and depleting natural resources, threatening their sustainability, and, thereby, undermining the resilience communities [4]. The resultant challenge requires transdisciplinary and transformative approaches in resource management, development and utilisation, using integrative approaches such as the water–energy–food (WEF) nexus, which allows for inclusive and equitable development, as well as coordinated resource planning and management [5]. 

The WEF nexus refers to the complex and inextricable interlinkages (synergies and trade-offs) that exist between water, energy, and food sectors, in pursuit of balanced and sustainable development. The WEF nexus approach takes into consideration cross-sectoral synergies and trade-offs among WEF sectors, while simultaneously mitigating duplication of activities. Development, management and use of resources through the WEF nexus is evidence-based, integrative, and ensures improvements in livelihoods and sustainability of resources for human well-being [6]. The current silo driven disproportionate use and allocation of WEF resources has created an imbalance in the physical and natural systems, to the extent that sustainability for future generations cannot be guaranteed [7,8]. For example, agriculture accounts for 70% of total global freshwater withdrawals, making it the largest consumer of water [9]. Water is used for agricultural production, forestry and fisheries, yet the same water is also required to produce or transport energy in different forms [9]. Concurrently, the food production and supply chain consumes about 30% of total energy consumed globally [10]. Continuing with the current siloed sectoral approach in resource management directly impacts on the livelihoods of the rural poor, and severely exacerbates their vulnerabilities as they generally lack resources to adapt to change [11].

Livelihood refers to the ability to obtain the basic needs in life, which include food, water, energy and clothing [12]. The sustainable rural livelihoods framework is the widely used approach in livelihoods studies, and emphasises on how people use their assets (natural, physical, social, human, and financial) to come up with livelihood strategies and positive outcomes [13]. In a livelihood approach, a detailed analysis of the factors that shape water, energy, and food security is done at local or community levels [1]. As livelihood approaches capture the processes and contextual factors that shape adaptive capacity, the WEF nexus analytical livelihoods framework can, thus, be integrated into livelihood analyses, as this framework is capable of assessing, monitoring and evaluating resource use and social development [14]. In most rural areas of developing countries, these three basic needs, and their delivery by government, are limited, which results in rural populations turning to natural systems for basic survival, or emigrating from rural to urban areas [15]. As the WEF nexus is mainly concerned with integrating these three connected resources of water, energy, and food, as well as simplifying human understanding of the complex linkages among them, and at the same time, ensuring resource security for sustainability; the approach is envisaged to provide pathways that transform rural livelihoods and ensure their resilience [14,16].

In the case of southern Africa, 60% of the population still lives in rural areas with limited access to these basic needs [6]. As land is a readily available resource, people in rural areas have always been largely dependent on farming as the main livelihood activity. Seventy-five percent of the income of rural households in southern Africa is derived from small-scale farming [5] under rainfed agriculture, coupled with a poor resources base, exposes rural populations to climate variability and change [17,18]. Other sources of income for people living in rural areas include non-farming natural resource based activities such as hunting and artisanal work, livelihood activities that are also climate sensitive [19]. All these factors contribute to the vulnerabilities of rural populations to the vagaries of extreme weather events and diseases [6].

Presently, almost 240 million people in sub-Saharan Africa (SSA) (or one person in every four) do not have access to nutritious food that guarantees a healthy and active life [20,21]. Rising food prices, and the recurrence of extreme weather events like floods and droughts, are pushing more people into poverty and hunger, compromising human health and well-being [22]. Africa’s population is anticipated to double to 2.4 billion by 2050, and already 38% of the people live in water scarce environments, as two-thirds of the continent is either arid or semi-arid [23,24]. Smallholder farmers who rely on rainfed agriculture contribute about 90% of the agricultural produce [25], yet the level of food insecurity in the region is very high, affecting mostly rural populations [26]. Dependence on climate sensitive sectors by small-scale farmers who contribute the most to the agricultural produce, but lack of access to resources to adapt is the major driver of food insecurity in the region. As climate change is cross sectoral and multidimensional, the WEF nexus can play an important role in climate change adaptation as it offers cross-sectoral mitigation and adaptation opportunities to harmonise interventions and build resilience [5].

The African Union Commission (AUC) through the Comprehensive Africa Agriculture Development Programme (CAADP) has earmarked to increase agricultural land by (i) extending area under sustainable land management and reliable water control systems, (ii) improving rural infrastructure and trade-related capacities for improved market access, (iii) increasing food supply and reducing hunger, and (iv) conducting research and development [27,28]. Implementing the WEF nexus enhances opportunities to achieve these targets as the approach can be used as a guide in decision making by assessing intervention mechanisms and balancing resource allocation to ensure equitable resource distribution [14]. For example, it suggested that increasing the area equipped with irrigation could provide opportunities for farmers to sustainably increase yield and address food insecurity, and CAADP aims to increase the irrigated area to 20 million ha, at an estimated initial cost of US$37 billion with infrastructure operation and maintenance requiring a further US$31 billion [27]. Whilst this huge investment is capable of transforming the agriculture sector, the question that immediately arises is, “where is the water to meet these set targets?”

Although emphasis on large-scale water infrastructural projects may have apparent beneficial outcomes like producing hydropower and providing water storage for irrigation and urban uses, such investments may be at the expense of downstream agro-ecological systems with adverse social consequences, such as displacement or resettlement [29]. In this context, this study adapted the WEF nexus analytical model developed by Nhamo et al. [14] to develop a WEF nexus analytical livelihoods framework (ALF), which was used to analyse and address the complex and interrelated nature of resource systems [14]. The WEF nexus ALF is a systems approach that analyses and assesses the interactions between the natural environment and the biosphere, facilitating a more coordinated management and monitoring of resources [5]. As the WEF nexus ALF monitors the performance of resource development, it is also essential for assessing performance and progress of sustainable development goals (SDGs) that are directly related to the WEF nexus (goals 2, 3, 6 and 7). The WEF nexus analytical model was used to assess resource utilisation and performance in southern Africa, in the context of achieving the 2030 global agenda of SDGs, that is, how best to build resilient rural communities and enhance sustainable rural livelihoods.

## 2. Materials and Methods 

### 2.1. The Study Area

**Figure 1:**
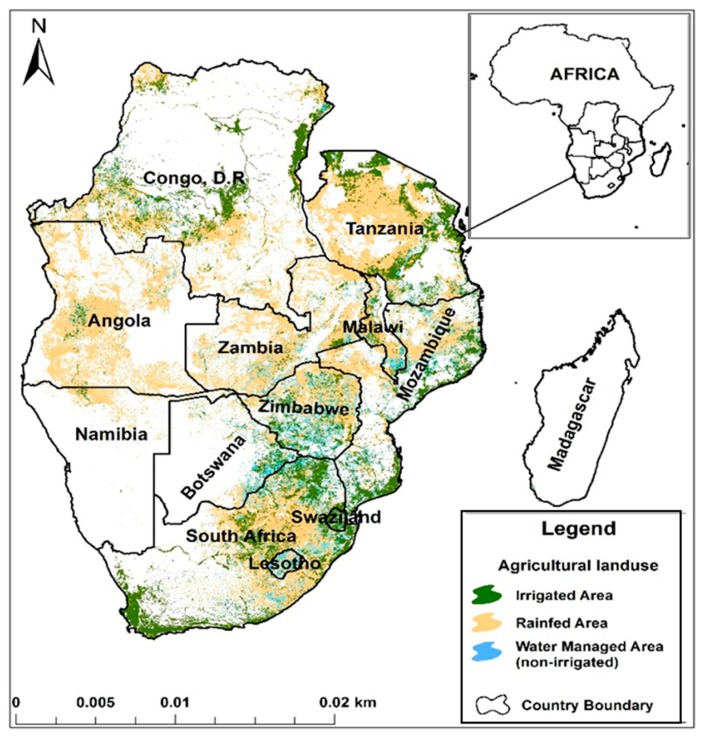
Locational map of southern Africa showing the distribution of agricultural systems. Cultivation is concentrated to countries in the east, whereas to the west, Botswana and Namibia have the least cultivated land due to arid conditions. Source: Adapted from International Water Management Institute (IWMI) irrigated area mapping (IWMI, 2010).

The study focused on southern African countries, namely Angola, Botswana, Comoros, Democratic Republic of Congo (DRC), Lesotho, Madagascar, Mauritius, Malawi, Mozambique, Namibia, Seychelles, South Africa, Swaziland, Tanzania, Zambia and Zimbabwe (Figure 1). The same 16 countries form an economic regional bloc known as the Southern African Development Community (SADC). Agriculture is the main source of livelihoods for the generally rural population, contributing about 17% to regional GDP (increasing to above 28% when middle income countries are excluded [30,31]. The SADC treaty, the overarching regional policy framework that aims to achieve economic development, peace and security, alleviate poverty and improve the livelihoods of the people, through regional integration [32]. To strengthen its agrarian economy, the region intends to increase agricultural production by increasing the irrigated area. Increasing agricultural production is envisaged to result in surplus yields that households of comparative advantage are able to supply regions of less potential, thereby ensuring food security and improving livelihoods. In this regard the region intends to double the irrigated area from 3.5% to 7% by 2025 [31].

Although irrigation potential in southern Africa is very high, agriculture remains rainfed (Figure 1). Land with irrigation potential is about 20 million ha, yet only 3.9 million ha is equipped for irrigation, accounting for approximately 6.6% of the total cultivated area [18,33]. Figure 1 also shows the distribution of agricultural systems in the region. The vision to increase the irrigated area targets the smallholder farming sector because of its importance in food security, but at the same time its high vulnerability to climate variability and change. Despite the huge agriculture potential and a big domestic market for agricultural products, poverty levels remain high, particularly rural areas [5]. Regional population stands at 342 million, of which 70% live in rural areas [34].

### 2.2. The Methodological Framework

**Figure 2:**
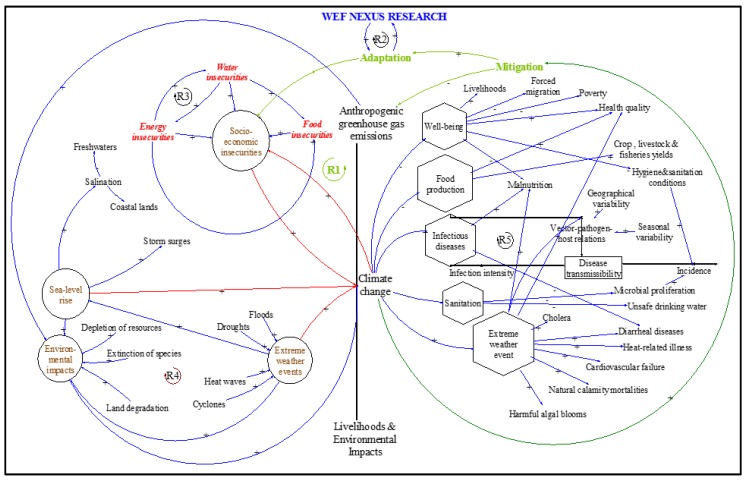
A systemic representation of the water–energy–food (WEF) nexus analytical livelihoods model showing the multi-dimensionality interactions and feedback effects of the system. The left enclosure shows measurable system components (encircled) as related to climate change. The right enclosure shows the impact of climate change (diamond-shaped) with vicious cascading effects on livelihoods and health.

Managing water, energy, and food resources as an interconnected system, and eliminating the traditional silo-based planning, improves sustainable development through research-based evidence [35,36,37]. The basis of the WEF nexus approach to resource management lies in identifying and justifying the interactions at the multiple interfaces of resource systems, while holistically assessing the impact of specific contexts or interventions from the environmental, financial, and socio-cultural perspectives [38]. It is from this systems perspective that Figure 2 is presented as a methodological framework, focusing on the improvement of rural livelihoods and health in the advent of climate variability and change. Figure 2 represents the components of the WEF nexus analytical livelihoods framework (ALF), as viewed from a systems thinking perspective. The WEF nexus ALF is adapted from the work done by McMichael et al., (2006), which depicts the pathways by which climate change can affect population health. Several climatic-environmental manifestations of climate change are shown, which altogether portray an increase in complexity of causal processes having environmental consequences and, therefore, the likelihood that health effects will be exacerbated or protracted. In order to elicit the linkages among the causes and the effects of climate, we converted the McMichael’s schematic summary (see Supplementary Material, Figure S1) of the main pathways by which climate change affects population health in to a causal loop diagram (CLD) shown in Figure 2. The systems approach integrates water, food, and energy aspects by mapping key elements of the subsystems and visualising their interdependencies.

The aim is to build livelihoods resilience through the WEF nexus by considering the linkages between WEF resources and causal mechanisms [39]. Improving livelihoods and social equality requires inclusive participation by all in decision making through consultation [40]. Faced with the realities of climate change, and how they are impacting mainly the rural poor, there have been loud calls to reduce greenhouse gas (GHG) emissions, which is the major cause of accelerated climate change [41,42]. For example, the Paris Climate Agreement brings the globe into a common cause of undertaking ambitious efforts to combat climate change and adapt to its effects, by keeping a global temperature rise this century well below 2 °C above pre-industrial levels, and to assist developing countries to achieve climate change adaptation [43,44].

The first part of the study relates to the impacts of climate change on WEF resources and proposes mitigation strategies through the WEF nexus ALF. We identify that, as people in rural areas generally depend on natural systems for their livelihoods, availability and accessibility to land and other natural resources are crucial for their survival. Then, secondly, the study demonstrates how the WEF nexus can be used as a decision support tool in addressing the pertinent issues, mainly transforming livelihoods and sustaining the environment. The premise is that resilience and adaptation must be decoupled from extensive use and depletion of natural resources and environmental damage, and promote sustainable means of production, investment, and consumption, along with enhanced resource efficiency and the reduction of waste, food losses, and pollution [45]. Adaptation through the WEF nexus ensures that vulnerable and marginalised populations are empowered through the removal of barriers that hinder balanced resource sharing and inclusive economic development [6]. Specific challenges brought about by climate variability and change, such as extreme weather events, resource depletion, among others (Figure 2) are addressed by strengthening resilience at the local and community level. Thus, the first and second parts of the methodological framework (Figure 2) emphasise both mitigation and adaptation. Mitigation is tackling the root cause of climate change to alleviate further damage to the atmosphere, and adaptation is dealing with the impact of climate change, such as extreme weather events and depletion of resources [46].

The third part addresses health effects of climate changes (Figure 2), and applies the WEF nexus analytical livelihoods framework to understand the causes and effects on human well-being, and to develop guidelines to alleviate health effects brought about by climate change. Environmental sustainability and human well-being are linked to the conservation of nature and natural resources and the preservation of biodiversity and ecosystems services and the WEF nexus plays an important role of guiding decision making [47,48]. Sustainable use and management of natural resources through the WEF nexus ensures water, energy and food security, equitable supply of public goods and services and inclusive development [5]. Thus, sustainable livelihoods transformation cannot be separated from climate change adaptation and the WEF nexus based research, as the approach is a “fitting tool” in transforming livelihoods, providing evidence to policy and decision-making in adaptation strategies.

The CLD shown in Figure 2 explains the WEF nexus analytical livelihoods framework and provides an understanding of the complex interrelationships among resources for livelihoods transformation, human health and well-being. The CLD provides a visual representation of multiple, interdependent effects of climate change and human actions on WEF resources. The Vensim PLE x32 software (www.ventanasystems.com) was used to develop the CLD for the WEF analytical livelihoods framework. The CLD is a qualitative representation of the interlinkages between resources utilisation and management and their effects on livelihoods in the advent of climate variability and change. The arrows show the influence of one variable on another (a change in the cause leads to a change in the effect). The polarity of the arrows indicates the factual relationship between any two nodes, which illustrate the causal link. The interplay between balancing and reinforcing loops gives rise to a realistic multi-loop system that explains the behaviour of the WEF model and the nexus impact over time. The reinforcing loop, R1, indicates the pathway through which mitigation measures lead to adaptation, with reduced anthropogenic GHG emissions in an amplified manner between resource insecurities and climate change. R1 is the ultimate loop that the WEF nexus research has to achieve. One of the means to achieve this aim is through the ability to assess the nexus interactions (evidence-based interventions) with amplifying effects for adaptation as in R2, which is the aim of this study. Other vicious bottlenecks in the system include R3 (the vicious reinforcing interrelationship between water, energy, and food insecurities), R4 (environmental behavioural patterns that contribute to climate change) and R5 (heightened disease transmissibility pathways).

The CLD justifies the context of complexity of causal mechanisms within which climate change occurs and the resulting causal effects it generates on the WEF resources, livelihoods, and health. It sets the context for considering the indicators listed in Table 1 for the pairwise comparison.

### 2.3. WEF Nexus Sustainability Indicators

Table 1 presents the WEF nexus sustainability indicators and their pillars that are considered when determining the WEF nexus composite indices [14]. Sustainability indicators for the WEF nexus performance are related to accessibility, self-sufficiency, availability, and how they influence respective production (productivity) [14]. Productivity, accessibility, self-sufficiency and availability are the major drivers of water, energy, and food security from where indicators are defined. Derived composite indices are important for assessing resource performance and management at any given scale, providing policy and decision making with valuable information on priority areas needing intervention [14]. Thus, the use of sustainability indicators and indices is an invaluable evaluation tool of the state of resources, either in the short and/or long term, providing directions for the actions to take in an attempt to ensure resources are sustainable, and, at the same time, improving livelihoods and well-being [14].

### 2.4. Overview of the WEF Nexus Indicators for Southern Africa

The importance of the WEF nexus is to simplify human understanding of the complex relationships among the interlinked resources of water, energy, and food, explain those relationships through quantitative evidence, and ensure the security of the three resources [14]. Factors that measure resource security include availability, accessibility, self-sufficiency, and productivity, from which we defined related indicators to measure the performance of the WEF resources [14,49]. Table 2 lists the defined indicators for the WEF resources, also providing an overview of the current status of the WEF nexus related indicators for southern Africa [50]. The selection of the indicators is based on the criteria used by Nhamo et al. [14] in which they considered the factors that determine resource security.

A multi-criteria decision method (MCDM) was used to integrate and establish numerical relationships among the WEF nexus indicators and calculate indices, applying the analytic hierarchy process (AHP), which is an MCDM method [51,52]. The AHP, developed by Saaty [53], is a theory of measurement for deriving ratio scales from both discrete and continuous paired comparisons to set priorities and make the best decisions. The AHP comparison matrix is determined by comparing two indicators at a time using Saaty’s scale, which ranges between 1/9 and nine [52]. A range between one and nine represents an important relationship, and a range between 1/3 and 1/9 represents an insignificant relationship. A rating of nine indicates that in relation to the column factor, the row factor is nine times more important. Conversely, a rating of 1/9 indicates that relative to the column indicator, the row indicator is 1/9 less important. In cases where the column and row indicators are equally important, they have a rating of one. In the case of the WEF nexus, the index of an indicator in relation to others is determined by the impact of that particular indicator on its overall rating.

With help of expert advice, the indicator status given in Table 2 provides the basis to establish numerical relationships among the indicators through a pairwise comparison matrix (PCM) as proposed by Nhamo et al. [14]. Using the AHP, the pairwise comparison matrix (PCM) is normalised to establish the composite indices for each indicator and an integrated WEF nexus index [14]. The indicator values shown in Table 2 are not disaggregated between rural and urban areas, as they represent the whole of the southern Africa region. However, it is common knowledge that rural areas have always lagged behind urban areas, in as far as economic development, provision of basic services, and access to resources is concerned [54]. Therefore, if the performance of resources used were classified as unsustainable, it would mean a worse situation in rural areas.

### 2.5. Determining the Pairwise Comparison Matrix and Normalisation of Indices 

Through the PCM, the AHP calculates the indices for each indicator by taking the eigenvector (a vector whose direction does not change even if a linear transformation is applied) corresponding to the largest eigenvalue (the size of the eigenvector) of the matrix, and then normalising the sum of the components [55]. The eigenvalue method synthesises a pairwise comparison matrix to obtain a priority weight vector for several decision criteria and alternatives. Here, an eigenvector of matrix is used for the priority weight vector. In the eigenvector method, the priority weight vector is set to the right principal eigenvector *w* of the pairwise comparison matrix [56]. The overall importance of each indicator is then determined. The basic input is the pairwise matrix, *A*, of *n* criteria, established on the basis of Saaty’s scaling ratios, which is of the order (*n x n*) [57]. *A* is a matrix with elements *a_ij_*. The matrix generally has the property of reciprocity, expressed mathematically as:

(1) $$a_{ij}=\frac{1}{a_{ij}}$$

After generating this matrix, it is then normalized as a matrix *B*, in which *B* is the normalized matrix of *A*, with elements *b_ij_* and expressed as:

(2) $$b_{ij}=\frac{a_{ij}}{\sum_{j=1}n\;a_{ij}}$$

Each weight value *w_i_* is calculated as:

(3) $$w_{i}=\frac{\sum_{j=1}n\;b_{ij}}{\sum_{i=1}n\;\sum_{j=1}n\;b_{ij}},\;i,j=1,2,3\ldots ,\;n$$

The integrated WEF nexus index is then calculated as a weighted average of all the indices of indicators. The integrated composite index represents the overall performance of resource development, utilisation, and management, as seen together.

### 2.6. Claculating the Consistency of the Pairwise Comparison Matrix

The consistency ratio (*CR*) is an indicator of whether the matrix judgments were randomly produced and that they are consistent [58]. Allowable *CR* values are those from 0.10 (10%) and below. Higher *CR* values indicate that the comparisons are less consistent, while smaller values indicate that comparisons are more consistent. When *CRs* are above 0.1, the process has to be repeated [52]. The *CR* is calculated as [59]:

$$CR=\frac{CI}{RI}$$

where: *CI* is the consistency index, *RI* is the random index, the average of the resulting consistency index depending on the order of the matrix is given by Saaty [52]. *CI* is calculated as:

$$CI=\gamma -\frac{n}{n-1}$$

where: λ is the principal eigenvalue (shaded section of Table 3), and *n* is the number of criteria or sub-criteria in each pairwise comparison matrix.

## 3. Results and Discussion

### 3.1. Impacts of Climate Change in Southern Africa

**Figure 3:**
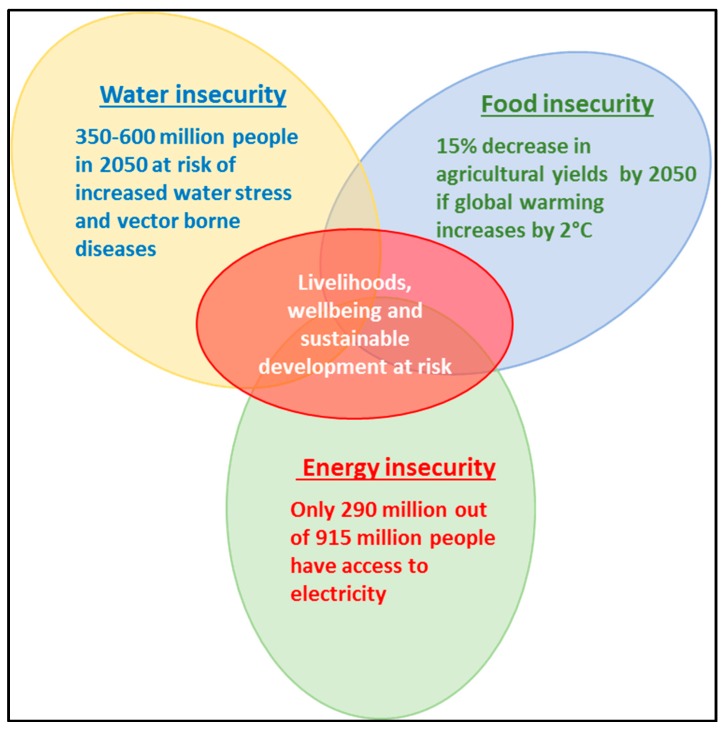
Summarised highlights of projected climate change risks on WEF resources for sub-Saharan Africa as highlighted in the text.

Southern Africa is highly vulnerable to climate change and variability because of multiple factors, such as reliance on climate sensitive sectors of agriculture and fisheries, lack of resources to adapt, poor infrastructure, and lack of institutional arrangements, as well as low adaptive capacity [60,61,62]. Water, energy and food are expected to be the sectors most affected by climate variability and change in the region [6]. The largest proportions of populations vulnerable to the vagaries of climate change are found on the African continent, where chronic water, food and energy insecurity and malnourishment remain endemic [5]. Rainfall variability threatens the production from more than 80% of agricultural land of the continent, as agriculture is mainly rainfed [17]. Climate projections for southern Africa indicate increased physical and/or economic water scarcity by as early as 2025 [63]. The Intergovernmental Panel on Climate Change (IPCC) estimates that between 75–250 million people and 350–600 million people in Africa will be at risk of increased water stress by 2020 and 2050, respectively [64] (Figure 3). Reduced rainfall, coupled with increased temperatures, will reduce (a) the area suitable for agriculture, (b) the length of growing period, and (c) yield potential [18,64]. By 2080, rainfall variability and longer dry spells would result in reduction of crop yields, rise in sea levels and coastal and low-lying areas would be affected by floods. Significant changes are already being experienced in sectors of agriculture, water, energy, biodiversity, and health [65] (Figure 3). Climate models predict that Africa will be able to provide only 13% of its food requirements by 2050 if no measures are in place to reduce GHG emissions [65]. Population is anticipated to have increased to 2 billion during the same period. Current statistics indicate that only 290 million out of 915 million people have access to electricity, and the total number without access is rising due to increased population growth and lack of contingency plans to improve the current situation [66]. Extreme weather events as caused by climate change derail progress made so far in poverty alleviation, employment, housing, access to and provision of services, food security and potable water [61].

Climate change will also result in an increase in vector borne diseases (Figure 3) such as malaria, dengue fever, yellow fever, among others [6,67]. Health challenges caused by climate change will be highly noticeable in regions experiencing extreme weather events like heatwaves, floods, storms, and fires. Other health risks resulting from climate change will be from changes in regional food yields, disruption of fisheries, loss of livelihoods, and population displacement due to sea-level rise, water shortages, loss of agricultural land, among others [68]. Water, energy and food security are closely related to health, as food and water, for instance, have a direct impact on human health and the physical conditions of humans has a strong influence on their ability to work [26].

Climate change models project that warming over the African continent is occurring at twice the global rate [65,69]. Without actions to reduce greenhouse gas emissions (GHG), temperatures are projected to rise more than 4 °C in southern African by 2100. Using the 1981–2000 base period, heatwaves have increased by over 3.5 fold to-date in the region [70,71]. Such changes in climate regimes, coupled with increased frequency and intensity of floods and droughts, are usually accompanied by health issues. The effects of extreme weather events transcends mortality and damage to property and crops, but also results in food and water insecurity, spread of disease, and mental health conditions [72].

### 3.2. WEF Nexus Analytical Livelihoods Framework

Adaptation strategies are wide-ranging in nature, and can be conceptualised broadly along a continuum, varying from interventions that are exclusively designed to mitigate the impacts associated with a changing climate, to initiatives aimed at addressing the wider underlying drivers of vulnerability and adaptive capacity [73]. Adaptation depends on the adaptive capacity of a household or community, which is generally lacking in most rural communities of southern Africa [62,74]. Adaptive capacity is the ability of a system to adjust to climate change and be able to accommodate shock or stress, or to expand the scope of variability with which it can cope [73]. The adjustments include controlling potential damage, taking advantage of opportunities, coping with consequences, as well as expanding coping range, and reducing vulnerability [73].

**Figure 4:**
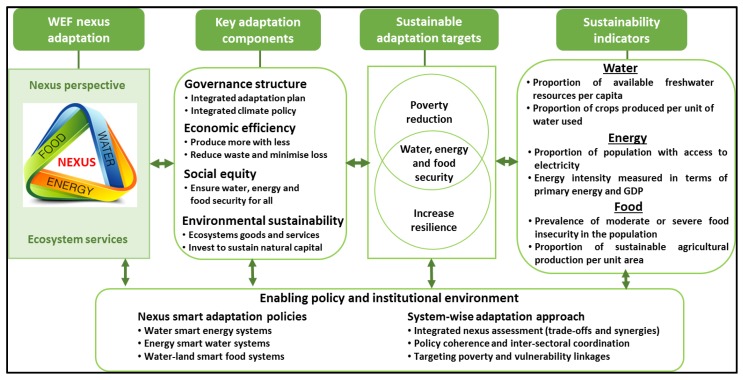
WEF nexus livelihoods adaptation and transformation framework.

Climate change adaptation refers to changes in processes, practices, or structures to offset potential damages, or to take advantage of opportunities associated with changes in the climate [75]. It encompasses actions that minimise the vulnerability of households, communities, and regions to climate change. Although adaptation can either be spontaneous or planned action, its success is determined by institutional and analytical frameworks that oversee and assess the level of vulnerability and the adaptive capacity [73]. The WEF nexus analytical livelihoods framework ensures an integrated management of resources, public services, and, at the same time considering synergies and trade-offs at all scales [76]. The transformation of rural livelihoods and the sustainability of adaptation strategies is underpinned by the understanding of the role of the WEF nexus in framing effective policies and institutions. Figure 4 represents a WEF nexus adaptation framework for assessing, monitoring and improving resource utilisation and management to ensure sustainable livelihoods transformation.

The first component of the framework (Figure 4) depicts the WEF nexus as a tool to enhance climate change adaptation and resilience for sustainable livelihoods and the environment [47]. It illustrates the intricacies in the interlinkages among the WEF nexus sectors. These envisaged outcomes are achieved through the key adaptation strategies of governance (policies and plans), social equity (accelerating access for all), environmental sustainability (investing to sustain ecosystem services), and economic efficiency (increasing resource efficiency), as shown in the second component [1]. These four key adaptation components form the basis to meet sustainable targets of reducing poverty and building resilience, which result in the security of resources, and sustainable development. The targets define sustainability indicators (last component of the framework) that assess and monitor resource planning and management, and to ensure equitable resource distribution and inclusive development. WEF nexus sustainability indicators are measurable parameters that indicate the performance of resource development, and monitor how the development is impacting on livelihoods or vice-versa [14]. The essence of the indicators is to connect statements of intent (objectives) and measurable aspects of natural and human systems. The four components of Figure 4 are supported and underpinned by an enabling environment that oversees the WEF nexus implementation [1].

### 3.3. Pairwise Comparison Matrix for WEF Nexus Indicators for Southern Africa

The PCM for the WEF nexus components for southern Africa is given in Table 3. The diagonal elements are the values of unity (i.e., when an indicator is compared with itself the relationship is one). Since the matrix is also symmetrical, only the lower half of the triangle is filled in and the remaining cells are reciprocals of the lower triangle. As already alluded to, the relationships are established using a scale ranging between 1/9 and nine as given by Saaty [52]. An overview of the regional indicator status for 2017 is shown in Table 2. The classification categories given in Table 5 also provides the basis of scaling the relationships. Thus, the indicator values given in Table 2 and their classifications provide the basis to classify the indicators.

### 3.4. WEF Nexus Composite Indices for Southern Africa

Composite indices for each indicator from the normalised PCM for southern Africa are indicated in Table 4, as calculated using Equation (3). Table 4 also provides the WEF nexus integrated index which is 0.145, a low index which reveals the unsustainability in performance of resource utilisation and management in the region, according to the classification by Nhamo et al. [14]. The CR for the pairwise matrix is 0.08, which is within the acceptable range. The weighted average of all the indices is the composite WEF nexus integrated index.

Table 5 provides the classification categories for the indicators as well as the WEF nexus integrated composite index for the ranking resource performance as proposed by Nhamo et al. [14].

### 3.5. Performance of WEF Nexus Indicators in Southern Africa

**Figure 5:**
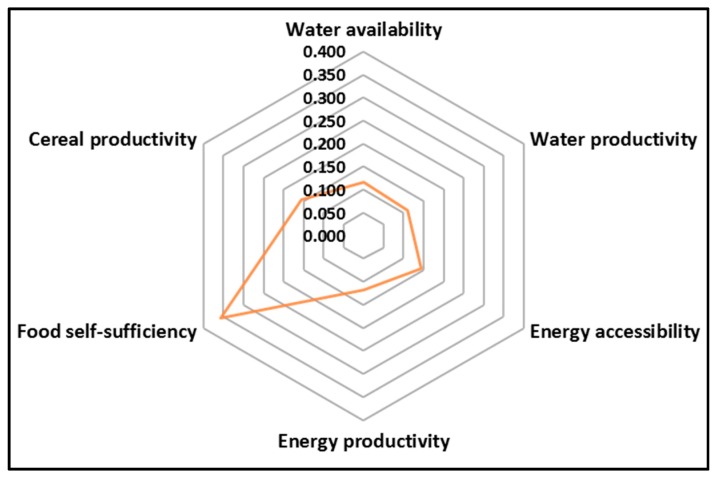
Current WEF nexus performance in southern Africa. The deformed amoeba (the orange centerpiece) is an indicator of an imbalanced and unsustainable resource performance, resulting from a siloed approach to resource use and management. The region should thrive to have a balanced centerpiece, which should be circular in shape.

Figure 5 is a graphical representation of the current performance and status of the WEF resources in southern Africa, showing a clear imbalance and uneven resource allocation and distribution. The lowly sustainable integrated WEF nexus index of 0.145 is an indication that the performance of resources utilisation and management is very low, and exacerbating regional vulnerabilities. There is an over emphasis on food security (food self-sufficiency) at the expense of other sectors. This is mainly due to sectoral approaches in resources management, which do not provide opportunities to minimise trade-offs. Although a lot of effort has been made on food self-sufficiency, showing an index of 0.357, water allocation towards agriculture is affecting other competing sectors. Most efforts seem to be directed towards the agriculture sector, yet water and hydropower (the main source of energy) are in short supply. Thus, Figure 5 pinpoints urgent sectors for prioritising interventions.

With the exception of South Africa, the region depends on hydropower as the main source of energy, and, thus, energy and agriculture compete for scarce water resources [77]. This places the region in a high vulnerable position because when there is drought, there is scarcity of water, energy, and food as these resources are climate sensitive, a three-way security threat. This was evident during the 2015/16 El Niño Southern Oscillation (ENSO) induced drought, which caused insecurities of WEF resources [62]. The increasing frequency and intensity of recent cyclones are causing immense devastation on agriculture, infrastructure, and loss of human life as well as human health [62]. Still under such an imbalanced situation, the region intends to increase the irrigated area from 3.5% to 7% of the cultivated area. Whilst increasing the irrigated area is a noble idea as it enhances food security to some extent, there is also the need to consider a cross-sectoral approach to avoid transferring challenges to other sectors, and, thus, to achieve a more circular shape in the WEF nexus indices in the spider graph (Figure 5). The shape of the spider graph indicates priority areas needing immediate intervention, i.e., all areas other than food self-sufficiency.

Under such a scenario as presented in Figure 5, the rural poor normally suffer the brunt of uncoordinated management of resources. Current resource management is unsustainably very low at 0.145. Although the region has improved agricultural production, as also indicated by previous studies [18], the region generally fails to meet the food requirements of its growing population (i.e., household food security), and usually relies on international aid to supplement local food deficits [62]. There is, therefore, a need for a paradigm shift from a sovereignty and silo mindset towards regional integration and cross-sectoral resources management for regional economic development, and to improve the livelihoods of people. This need is supported by the transboundary nature of resources in southern Africa, and similar challenges that span across countries, as climate change does not respect political boundaries [5].

The WEF nexus analytical tool is important for transforming livelihoods, as it identifies priority areas for intervention and ensures that rural areas are not left behind in regional, national, and local developmental projects. Prioritising rural development in southern Africa ensures gender and social inclusivity by improving the livelihoods of vulnerable groups such as women, children, and the youth who form the majority of rural communities [78,79]. The results indicate the need for regional planning, coordination and monitoring of resources in southern Africa, as resources are generally transboundary [6]. For southern Africa in particular, managing resources at a regional level is a catalyst for regional integration, and a pathway to improve rural livelihoods as well as attaining the 2030 agenda on sustainable development.

## 4. Recommendation on Improving Rural Livelihoods in Southern Africa

Improving livelihoods, particularly rural livelihoods, is dependent on the implementation of a holistic and systematic approach such as the WEF nexus analytical livelihoods framework. Whilst food and nutrition security are a priority, especially in southern Africa, a WEF nexus approach would mitigate trade-offs and highlight vicious feedbacks with energy and water. Importantly, the WEF nexus approach provides a framework for sustaining rural resources, whilst maximising on the positive synergies, and identifies how best to build resilience into rural livelihoods and wellbeing. The following recommendations are proposed for improving resource management and livelihoods of poor rural people in southern Africa in the face of climate change, and at the same time, pursuing regional development goals, in a manner that integrates across the region, which enhance synergies and emergent properties:

▪ Embracing a cross-sectoral nexus thinking in conceptualising, implementing and evaluating WEF resources planning and management. Specifically, this could be incorporated within a theory of change (TOC) model to establish the pathways to understanding the underlying logic, assumptions, influences, causal linkages, and expected outcomes of the WEF nexus analysis in uplifting rural livelihoods and building resilience to climate change.
▪ Exploit the untapped abundant renewable green energy sources, like wind and solar, to increase energy availability and access, as well as to reduce energy costs and providing clean energy for poor rural people. Solar and wind energy can easily reach the rural population as they can be installed near the demand area, further reducing the environmental footprint and the unintended trade-offs and/or consequences for other sectors, such as water security or food production.
▪ Explore conjunctive water use, through exploiting untapped groundwater resources for irrigation and domestic use to counter rainfall variability, and to supplement surface water resources. Currently, 80% of the cultivated area is rainfed, which is at great risk from climate change, and local access to groundwater resources to supplement rainwater, would enhance both household food security and household water security.
▪ A combination of increased access and availability of water, combined with clean energy, would contribute significantly to uplifting the standard of living (nutrition, health, and well-being) among poor rural people, whilst countering the potential negative consequences of climate change on these. This is especially so for public health elements associated with malnutrition, water-borne diseases, and food preparation and heating with wood and paraffin.
▪ Design and develop cross-sectoral governance structures at regional level like climate change policies, strategies, and adaptations plans. These should be aligned to regional institutions and policies to unlock the potential of the WEF nexus approach, and to effectively exploit the potential of transboundary resources. For example, the Regional Agriculture Policy (RAP) can be linked to the Water and Energy Policy, such that they can inform decision making as on sustainable agricultural expansion and development, reveal trade-offs across sectors, and reduce unintended consequences, especially for downstream users.
▪ Investments in research, development and innovation, which promote the production of more with fewer resources, the reduction of waste, and minimising losses, for enhanced sustainability. This includes investments in efficient energy technologies to improve energy use efficiencies and productivity. Secondly, investments in smart and efficient irrigation technologies, including for local groundwater and storage capacity, would contribute to decreasing agricultural surface water use, and improved water productivity and access, and resilient household agricultural production. In addition, adoption of climate and water smart agricultural practices could reduce water and energy demand in agriculture.
▪ Build resilience at the regional level and promote integration through the WEF nexus approach as it identifies opportunities for climate change adaptation and reduction of poverty and vulnerability through coordinated WEF infrastructure development, improved management of transboundary natural resources and maximising on regional competitive advantages for agricultural production. This has potential to improve regional resilience and resource use efficiency. This is particularly relevant for southern Africa as most resources are transboundary and, if not well managed they may become a cause of conflict [5].
▪ As the WEF nexus is at the heart of sustainable development, it is also central to regional dialogues on economic development and monitoring the performance of SDGs. Therefore, adopting the WEF nexus at the regional level promotes sustainable resource utilisation and inclusive economic development and, job creation, thereby improving the livelihoods and well-being of people. The WEF nexus is, thus, a tool capable of assessing and monitoring SDGs implementation and performance, particularly SDGs 2, 3, 6 and 7. For example, there is a strong case for a regional food security initiative as opposed to current national food security strategies, as this would maximise the region’s competitive advantages in crop and livestock production, whilst maximising household food security, and buffering the region against local droughts. This would also encourage new investments in areas that currently lack any, leading to job creation, and creating a virtuous circle for regional integration efforts.
▪ Link climate change scenario planning with the WEF nexus analytical livelihoods framework to enhance the reflexivity, resilience, responsiveness, and revitalisation of governance and adaptation strategies. Reflexivity is the capability to systematically and continuously deal with a variety of problems as they emerge; resilience is the ability to bounce back to the original basic state of function after a perturbation; responsiveness is the ability to deal with dynamic demands and expectations, and; revitalisation is the ability to reignite policies and ensure their continuous application [80]. These scenario planning include the shared socioeconomic pathways (SSPs) and the representative concentration pathways (RCPs), which are a set of pathways and frameworks developed to facilitate an integrated analysis of long-term and near-term modelling experiments for climate change to assess vulnerabilities and recommend adaptation and mitigation strategies [81,82].

## 5. Conclusions

The development of the WEF nexus indicators and composite indices for southern Africa has highlighted the unsustainability of current resource utilisation and management in the region; this is mainly due to the siloed sectoral approach to resource management. Whilst more emphasis on food self-sufficiency may ensure national food security to some extent, there are risks to rural household food security and well-being, and transferring problems to other sectors when there is no balance and coordination in resource development and utilisation among sectors. The WEF nexus analytical livelihoods framework is a catalyst for managing resources and building resilient rural communities in a sustainable manner as it indicates priority areas needing immediate intervention. The numerical relationship among the WEF nexus indicators show an unbalanced resource management, a situation that increases risk and vulnerability. The transboundary distribution of resources in southern Africa favours the management of resources at a regional level; this would contribute to the region’s developmental targets and to achieving the 2030 Global Agenda on Sustainable Development. The study demonstrates the use and importance of the WEF nexus in providing decision makers with tools to holistically and systematically develop, manage and monitor resource use for sustainable development. We highlight the potential of the WEF nexus as an inclusive, transparent, intergovernmental approach for all stakeholders, supporting SDGs as well as promoting the formulation and use of scientifically enabled policies, monitoring, assessment, and cooperation. The link between the WEF indicators and SDGs indicators is an opportunity for further studies on assessing the performance of related SDGs using the WEF nexus analytical model. The tool is important for climate change adaptation strategies and for creating resilient communities through the coordinated resource management framework presented in the study. The analyses highlighted the gap in data availability at the household level, thus this study focused on regional level analyses. Future studies should focus on the household scale analyses as this will translate to greater impact. Most critically, our WEF nexus approach emphasises the trade-offs and unintended consequences of the current approaches, with direct negative consequences for poor rural households in terms of resource security and well-being, but, at the same time indicating priority areas for intervention.

## Figures and Tables

**Table 1:** Indicators and sub-indicators for the WEF nexus component.

WEF Nexus Component	Indicator	Pillars
1. Water	Proportion of available freshwater resources per capita (availability) Proportion of crops produced per unit of water used (productivity)	Affordability Stability Safety
2. Energy	Proportion of population with access to electricity (accessibility) Energy intensity measured in terms of primary energy and GDP (productivity)	Reliability Sufficiency Energy type
3. Food	Prevalence of moderate or severe food insecurity in the population (self-sufficiency) Proportion of sustainable agricultural production per unit area (cereal productivity)	Accessibility Availability Affordability Stability

Source: Nhamo et al., 2019 [14].

**Table 2:** Overview of the WEF nexus indicators for southern Africa.

WEF Nexus Component	Indicator	Indicator Status 2017 *
1. Water	Proportion of available freshwater resources per capita (availability)	3984 m^3^
	Proportion of crops produced per unit of water used (water productivity)	$10/m^3^
2. Energy	Proportion of population with access to electricity (accessibility)	42.8%
	Energy intensity measured in terms of primary energy and GDP (productivity)	7 (MJ/GDP)
3. Food	Prevalence of moderate or severe food insecurity in the population (self-sufficiency)	8%
	Proportion of sustainable agricultural production per unit area (cereal productivity)	1395 kg/ha

Source: World Bank Indicators, 2017. * The reported indicators include both rural and urban populations.

**Table 3:** Pairwise comparison matrix for the WEF nexus indicators for southern Africa.

Indicator	Pairwise Comparison Matrix					
	Water Availability	Water Productivity	Energy Accessibility	Energy Productivity	Food Self-Sufficiency	Cereal Productivity
Water availability	1	1/3	1/3	1	1	1
Water productivity	3	1	1/3	1/3	1/3	1
Energy accessibility	3	3	1	1	1/5	1/3
Energy productivity	1	3	1	1	1/3	1/3
Food self-sufficiency	1	3	5	3	1	7
Cereal productivity	1	1	3	3	1/7	1

**Table 4:** Normalised pairwise comparison and composite WEF nexus indices for southern Africa.

Indicator	Water Availability	Water Productivity	Energy Accessibility	Energy Productivity	Food Self-Sufficiency	Cereal Productivity	Indices
Water availability	0.100	0.029	0.031	0.107	0.332	0.094	0.116
Water productivity	0.300	0.088	0.031	0.036	0.111	0.094	0.110
Energy accessibility	0.300	0.265	0.094	0.107	0.066	0.031	0.144
Energy productivity	0.100	0.265	0.094	0.107	0.111	0.031	0.118
Food self-sufficiency	0.100	0.265	0.469	0.321	0.332	0.656	0.357
Crop productivity	0.100	0.088	0.281	0.321	0.047	0.094	0.155
CR = 0.08							∑ = 1
Composite WEF nexus index (weighted average)							0.145

**Table 5:** WEF nexus indicators performance classification categories.

Indicator	Unsustainable	Lowly Sustainable	Moderately Sustainable	Highly Sustainable
Water availability (m^3^/per capita)	<1700	1700–6000	6001–15,000	>15,000
Water productivity (US$/m^3^)	<10	10–20	21–100	>100
Food self-sufficiency (% of pop)	>30	15–29	5–14	<5
Cereal productivity (kg/ha)	<500	501–2000	2001–4000	>4000
Energy accessibility (% of pop)	<20	21–50	51–89	90–100
Energy productivity (MJ/GDP)	>9	6–9	3–5	<3
WEF nexus composite index	0–09	0.1–0.2	0.3–0.6	0.7–1

Source: Nhamo et al., 2019 [14].

## Supplementary Materials

The following are available online at https://www.mdpi.com/1660-4601/16/16/2970/s1, Figure S1: Schematic summary of main pathways by which climate change affects livelihoods, human wellbeing and health, and how they are related to the WEF nexus. The pathways formed the basis to develop a systems approach that integrates water, food, and energy aspects by mapping key elements of the subsystems and visualising their interdependencies.
