# Supplementary material for: The Water–Energy–Food Nexus as a Tool to Transform Rural Livelihoods and Well-Being in Southern Africa
Source: Int J Environ Res Public Health. 2019 Aug 18;16(16):2970. doi: 10.3390/ijerph16162970 (PMC6720849; doi:10.3390/ijerph16162970)
Supplement: Supplementary file 1 [file ijerph-16-02970-s001.pdf]

Supplementary Material:

Main pathways by which climate change affects livelihoods

Tafadzwanashe Mabhaudhi, Luxon Nhamo, Sylvester Mpandeli, Charles Nhemachena, Aidan Senzanje, Nafiisa Sobratee, Pauline P. Chivenge, Rob Slotow, Dhesigen Naidoo, Stanley Liphadzi and Albert T. Modi

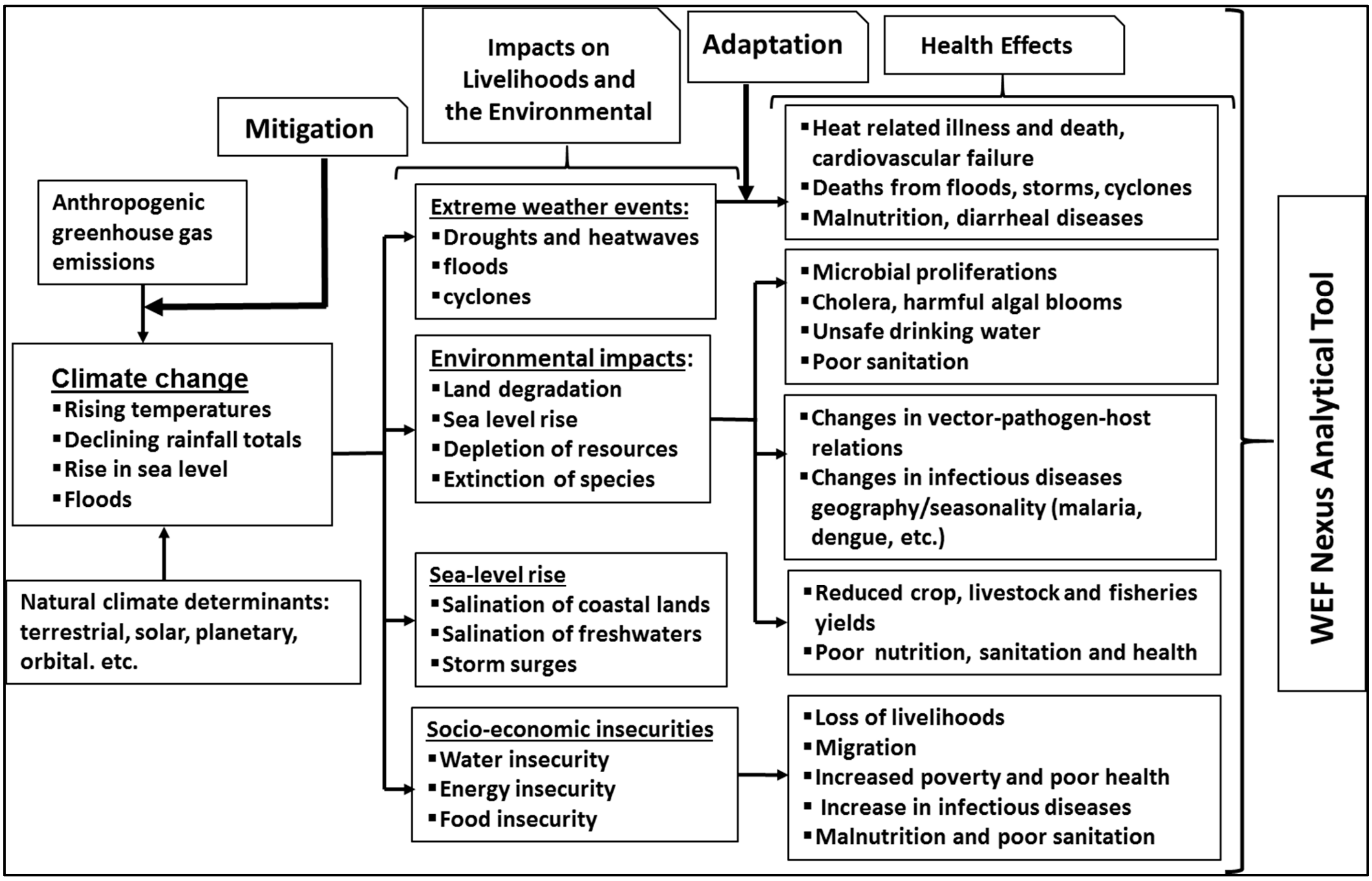

Figure S1. Schematic summary of main pathways by which climate change affects livelihoods, human wellbeing and health, and how they are related to the WEF nexus. The pathways formed the basis to develop a systems approach that integrates water, food, and energy aspects by mapping key elements of the subsystems and visualising their interdependencies.
Source: Adapted from McMichael et al., 2006

© 2018 by the authors. Submitted for possible open access publication under the terms and conditions of the Creative Commons Attribution (CC BY) license (http://creativecommons.org/licenses/by/4.0/).
